# Waste battery disposal and recycling behavior: a study on the Australian perspective

**DOI:** 10.1007/s11356-022-19681-2

**Published:** 2022-04-04

**Authors:** Md Tasbirul Islam, Nazmul Huda, Alex Baumber, Rumana Hossain, Veena Sahajwalla

**Affiliations:** 1 School of Engineering, Macquarie University, 44 Waterloo Road (44 WR), Sydney, NSW 2113 Australia; 2 Centre for Sustainable Materials Research and Technology (SMaRT Centre), School of Materials Science and Engineering, University of New South Wales (UNSW Sydney), Sydney, NSW 2052 Australia; 3 Faculty of Transdisciplinary Innovation, University of Technology Sydney (UTS), Broadway, Sydney, NSW 2007 Australia

**Keywords:** Spent battery, Consumer, Policy, Recycling, Circular economy, Extended producer responsibility

## Abstract

Consumer behavior is a critical consideration for the development of sustainable waste management systems, including waste batteries, which pose a serious threat to human health and the environment if disposed of improperly. This study investigates the consumers’ perspective on the waste battery collection and recycling behaviors in Australia, and analyses their implications for the development of recycling schemes. The results show that, although general awareness exists among consumers about the negative impacts of improper disposal, this awareness was not reflected during the disposal of waste batteries among the participants. Insufficient knowledge about the waste battery collection points and convenience were the most important factors affecting the inappropriate disposal behavior from most of the consumers. Over 50% of participants were unaware of the collection points for waste batteries. The most-preferred battery collection systems involved a deposit return system similar to that used for bottle recycling in the state of New South Wales (NSW) or collection at supermarkets/retailers. The most preferred methods for providing an incentive to recycle batteries were “old-for-new” battery swaps, “vouchers that could be used for other items in a store,” and “cash payments.” Several policy implications have been highlighted from this pioneering study that could shape the future development of sustainable waste battery management systems in Australia.

## Introduction

Batteries support many aspects of modern lifestyles due to their portability and diversified use in electrical and electronic equipment (EEE) and large industrial applications (e.g., battery storage system for solar PV panel), with global demand forecasted to continue rising by 7.8% per year (Carberry 2017). Batteries contain harmful elements such as cadmium, lead, and mercury, that are detrimental to human health and the environment if not properly managed (Sun et al. 2015). Various materials used in different battery types are shown in Table. 1. Household handheld batteries (HHB) are characterized by chemistry (or material) types and sizes. AAA, AA, 9 V, C, and D are some of the sizes, and alkaline, carbon-zinc, lithium, nickel-metal hydride, and nickel–cadmium are the chemistry types of HHB, respectively (ABRI 2010). Improper disposal of batteries into landfills is the greatest cause of heavy metal contamination (Krekeler et al. 2012). Aside from preventing contamination, battery recycling can recover metals such as lithium and cobalt for reuse in new battery manufacturing, reducing the environmental burden caused by mining new materials (Islam and Huda 2019, Oliveira et al. 2015). The presence of heavy and valuable metals makes waste batteries (WBs) one of the most important waste streams for the development of the circular economy (Porvali et al. 2020). Circular economy (CE) principles emphasize the importance of engaging consumers in the selection of appropriate options for end-of-life (EoL) management (Ellen MacArthur Foundation 2017).

**Table 1:** Material use in various batteries, adopted from (Australian Battery Recycling Initiative 2022)

Battery type	Acronym	Cathode material	Anode material	Electrolyte	Example recycling process/technology
Alkaline-primary (single use)		Manganese dioxide	Zinc	Aqueous alkaline or potassium hydroxide	Carbothermic process for recovering high-value zinc/zinc oxide (Zn/ZnO) powder (Mukhlis et al. 2021), catalysts-based total oxidation of hydrocarbons (acid treatment) (Park et al. 2021)
Lead-acid	ULAB	Lead dioxide	Lead	Sulfuric acid	Hydrometallurgical and pyrometallurgical processes (Yanamandra et al. 2022)
Li-Ion	Li-ion	Metal oxides of cobalt, nickel, iron, aluminum, or manganese	Carbon	Lithium salt in a solvent such as (organic, solid ceramic, ionic fluid, composites, or other types of solvent)	Mechanical treatment, pyrometallurgy, or hydrometallurgy (Sommerville et al. 2021)
Nickel–cadmium	NiCd	Nickel oxyhydroxide	Cadmium	-	Chemical recycling of cadmium (Saleh et al. 2021), pyro-and hydrometallurgical processes (Blumbergs et al. 2021)
Nickel metal hydride	NiMH	Nickel oxyhydroxide	Hydrogen-absorbing alloy	Potassium hydroxide	Hydrothermal, NMP dissolving, bioleaching treatment, acid leaching (Pradhan et al. 2021)

**Fig. 1:**
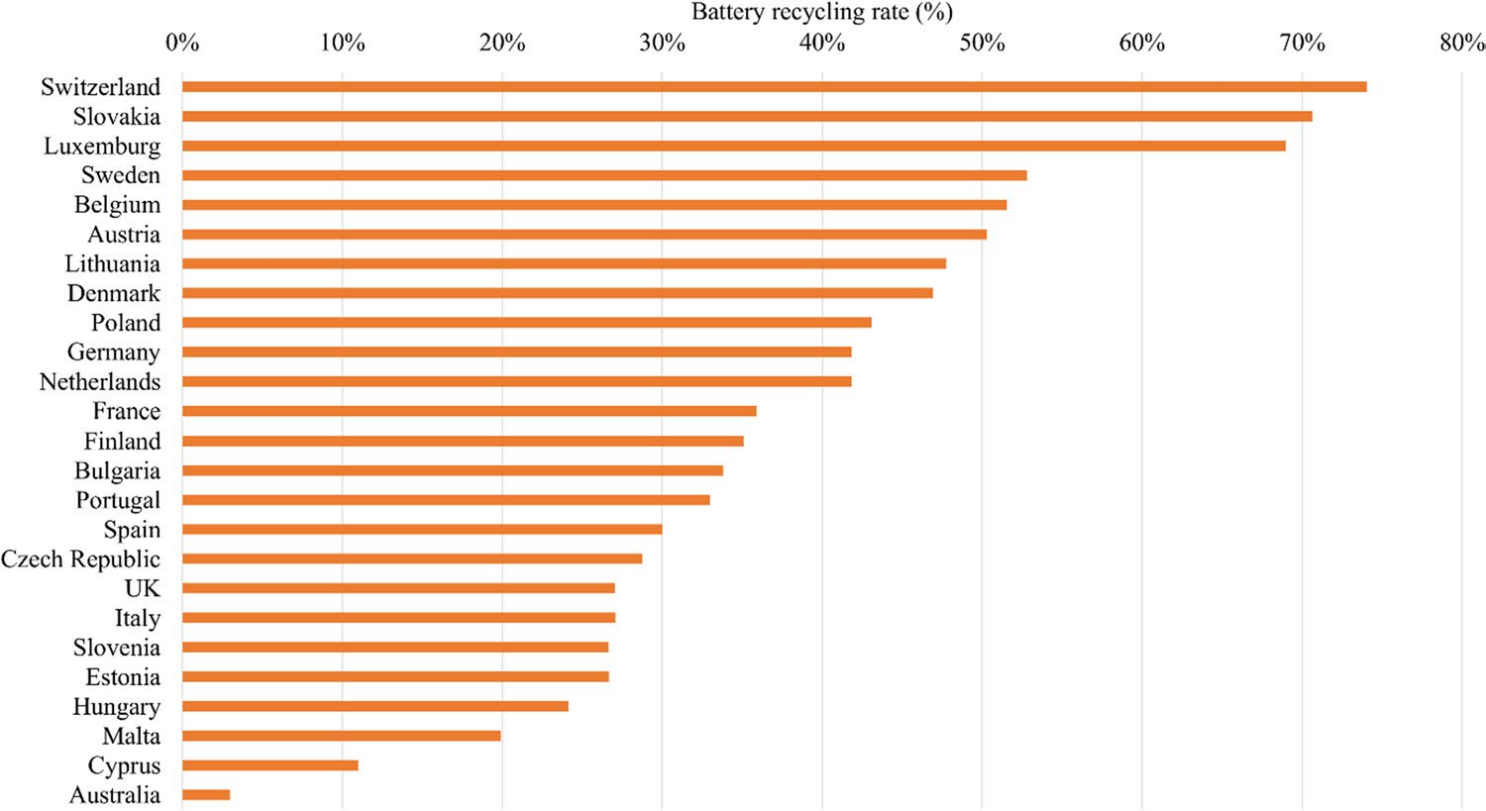
Battery recycling rate in OECD countries including Australia (ABRI 2017)

From material circulation and environmental impact mitigation perspectives, globally, numerous measures have been taken. European Union (EU) has already taken holistic steps such as proposing new battery regulation and sustainable battery management strategies for circular and climate neutral economy under circular economy action plan and European Green Deal (European Commission 2020a). The new proposed regulation critically stressed the issues of providing better and more reliable information and guidance on battery labeling system and battery performance, so that consumers can make an informed purchase decision and be part of the appropriate waste disposal mechanism. Thus, consumer behavior is found to be one of the influencing factors in the value chain (European Commission 2020b). Since 2006, in the EU, Batteries Directive (2006/66/EC) (European Commission 2006) has been enforced, which resulted in a higher collection and recycling rate. For example, 51% of the portable batteries sold in the EU were collected and recycled in 2019 (Eurostat 2022), which is aimed to increase to 65% in 2025 and to 70% in 2030 (European Commission 2020a). Some of the countries in the EU already reached that target (over 70% collection and recycling rate of waste batteries), such as Switzerland (Fig. 1) (ABRI 2017). The country is also one of the Organisation for Economic Co-operation and Development (OECD) countries. The success factors behind such achievements are (a) customers’ obligation disposing of WBs at dedicated collection points and (b) operational and financial sustainability (funding of the system) of the collection and recycling system by introducing advanced recycling fee (ARF), collected at the point of purchase. A direct interaction is being established with the consumers and producers of the batteries via the principles of extended producer responsibility (EPR) (i.e., producers are responsible for treating or disposing of post-consumer products (OECD 2019).

**Fig. 2:**
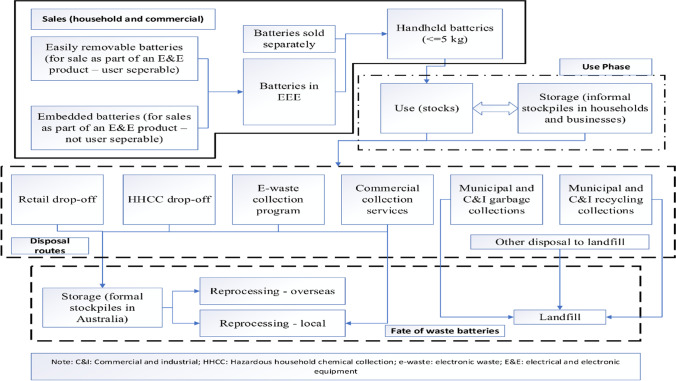
Battery use, (waste battery) collection and recycling routes in Australia, adapted from (Kyle O’Farrell et al. 2020)

Despite being one of the major strategic countries in the OECD, Australia’s progress in WB collection and recycling is very insignificant (O’Farrell et al. 2014). The industry-led Australian Battery Recycling Initiative (ABRI) is advocating for the development of “Battery Stewardship scheme” since its formation in 2008. ABRI consists of manufacturers, recyclers, retailers, government organizations, and environmental groups. In September 2020, the scheme was endorsed by the Australian Government. In Australia, “The Product Stewardship Act 2011” (one of the significant environmental strategies for making designers, producers, retailers, and consumers responsible for the entire product lifespan) identified all types of batteries as “priority products” (Salim et al. 2019). However, no specific regulations have been established regarding WB collection and recycling, to date (Salim et al. 2019). Only recently (in 2019), waste lithium-ion batteries (LIBs) recycling was included in the National Waste Policy Action Plan. This also shows the infancy of the progress in the WB management sector in Australia. HHBs and other types of batteries shown in Table 1 are largely processed by the scrap metal companies and then exported to countries such as China, as there is no dedicated recycling infrastructure or technology available (King and Boxall 2019). However, this trend has recently been impacted by the National Sword Policy of China, limiting the waste export and overseas processing (King and Boxall 2019), resulting in large-scale landfilling, causing a serious environmental problem (CSIRO 2022). In 2012, 97% of handheld batteries were disposed of in Australian landfills (O’Farrell et al. 2014). The current waste battery collection and recycling system is presented in Fig. 2.

Circular economy (CE) principles emphasize the importance of engaging consumers in the selection of appropriate options for end-of-life (EoL) management (Ellen MacArthur Foundation 2017).

A recent report by Commonwealth Scientific and Industrial Research Organisation (CSIRO) identified that improved understanding of the importance of recycling, collection process, and efficient way of material recycling are some of the critical steps to overcome the low waste battery recycling rate (CSIRO 2022). Customers are one of the key stakeholders under the Product Stewardship scheme, and in the battery supply chain. Their behavioral aspects are critical for waste product collection and recycling (Wagner 2013). The “Battery Stewardship scheme” envisioned by ABRI requires careful understanding of consumer behavior to divert WBs from landfill (ABRI 2017). The downstream efficiency of recycling programs largely depends on consumer behavior (King and Boxall 2019). Understanding general consumer perceptions regarding collection and recycling responsibilities may assist with the development of the scheme (ABRI 2017). Arbués and Villanúa (2016) mentioned that understanding consumers’ recycling behavior is particularly important in designing long-term planning for a recycling system.

Most of the Australian WB collection and recycling-related researches were focused on LIBs, for example, King and Boxall (2019) analyzed enablers and barriers developing a new LIB recycling industry. Zhao et al. (2021) focused on LIBS growth drivers, markets, and the status LIBs recycling and reuse specific landscape. Environmental impacts of LIBs recycling (Boyden et al. 2016), urban mining of LIBs (Boxall et al. 2018), and e-waste-specific battery recycling (Islam and Huda 2020b) have also been the recent areas of discussion. However, these studies have not conducted a consumer survey understanding their disposal and recycling behavior with a specific focus on HHBs. In the context of WB collection and recycling, this study aims to identify consumers’ disposal and recycling behavior focusing on HHB using a questionnaire survey.

To the best of the authors’ knowledge, this is the first systematic attempt to investigate consumer attitudes and behavior around WB collection and recycling in the Australian context. It aims to contribute to the global literature through an analysis of a context where most WBs currently end up in landfill, as well as to provide valuable information to policymakers for the design of future battery collection and recycling scheme in the country. The paper is organized as follows: Section 2 conducts an in-depth literature review. Section 3 discusses the research methodology of the study; Section 4 presents the results of the survey and discussions. Section 5 provides some useful future directions, and states research limitations of the study and finally, Section 6 reaches a conclusion.

## Literature review

Previous studies need to be analyzed to identify the scope of the study and design the survey. Using the keywords “waste battery” OR “spent battery” in the web of science (WoS) core collection database, 325 articles were identified. WoS database shows articles published in international peer-reviewed journals. As this is a consumer questionnaire survey study, the word “survey” was given in the refined search box of the database, resulting in 23 articles. Some of the articles were also collected from Google scholar search utilizing keywords, “waste battery,” “spent battery,” and “survey.” After the initial screening (title and abstract) and further analysis of the methodology section, only seven articles were identified those:

1. performed survey on consumers, residents, individuals, and others;
2. focused on any of the waste battery streams (lead-acid, lithium-ion battery, and others);
3. mentioned specific types of survey data collection techniques (e.g., online, face-to-face);
4. and showed a definite number of samples.

Only a few surveys related to studies have been published (mostly from China) focusing on WB collection and recycling. Associated WB collection and recycling regulations in the respective country were also given in Table 2. Table 3 shows the summary of the articles.

**Table 2:** Waste battery collection and regulations in selected countries

Country	Relevant and latest policy/regulation/legislation on waste battery collection and recycling in the respective country	Reference
China	The Law of the People’s Republic of China on the Prevention and Control of Solid Waste Pollution (2020 edition)	Sun et al. (2021)
Japan	The Law for the Promotion of the Effective Utilization of Resources (2000)	Australian Battery Recycling Initiative (2014)
Russia	No specific regulation on waste battery collection and recycling. “The Emergence of Responsible Waste Management 2019″ is the government policy that focuses on recycling	Fedotkina et al. (2019)
Spain	Royal Decree 106/2008 Of 1 February on Batteries and Accumulators And Their Waste Environmental Management	Arbués and Villanúa (2016)
Switzerland	a. Chemical Risk Reduction Ordinance (ORRChem); b. Ordinance on the prepaid disposal fee for batteries; c. Ordinance on Movements of Waste (OMW); d. DETEC Ordinance on Lists relating to Movements of Waste (LMW)	Federal Office for the Environment (2019)
Australia	No specific regulation available. Voluntary battery stewardship scheme was granted authorization by Australian Competition & Consumer Commission in September 2020. In 2013–2015, first the battery industry working group was formed	Australian Competition & Consumer Commission (2020)

**Table 3:** Summary of waste battery disposal and recycling-behavior focused questionnaire survey studies

Reference	Country (according to the first author)	Research focus	Level of analysis	Kind of batteries considered	Type of survey	Number of samples (effective)	Demographic characteristics considered	Application of statistical/analytical tools and techniques
Sun et al. (2015)	China	Battery collection and recycling	Residents	Lead-acid batteries and household batteries (dry batteries, LIBs, and button cells)	On-site survey	1874	Gender, age, occupation, level of education, monthly income, residents’ region	-
Gu et al. (2017)	China	Ownership, replacement, disposal, and recycling behavior	Individuals	LIBs	Online survey	786	Age, occupation, income/month, and residents’ region	Grey Verhulst model (GVM)
Chen et al. (2017)	China	Sales, use, recycling, and disposal pattern	Retailers, corporates, and individuals	Spent e-bicycle batteries (lithium batteries)	Face-to-face Interview	2138	Gender, age, level of education, and annual income	Canonical correspondence analysis (CCA) using R-package and SPSS
Tian et al. (2015)	China	WTP and recycling behavior	Residents	Lead-acid battery (LAB)	Face-to-face interview	931	Age, gender, education level, and household income	0–1 style logistic regression and contingent value method (CVM)
Asari and Sakai (2013)	Japan	Consumer behavior, recycling, and cobalt flow	Individuals	LIBs and small batteries (individually used or used in small electronic products)	Online survey	6500	Age	-
Tarasova et al. (2012)	Russia	Willingness to participate in the collection system	Residents	Household batteries (zinc/manganese dioxide batteries)	Not mentioned specific type	526	Place of residence (region), occupation, and age	-
Arbués and Villanúa (2016)	Spain	Waste battery collection	Households	Not specified	Personal interview	More than 20,000 (secondary survey data)	Income, age, working status, gender, education level	Bivariate probit model
Hansmann et al. (2006)	Switzerland	Recycling behavior, battery consumption	Individual participants	Not specified	Mail survey (paper based)	608	Age, gender, household size, family income, region of residence, language spoken	Analyses of covariance (ANCOVAs), regression analyses
Salim et al. (2019)	Australia	Drivers, barriers, and enablers of waste solar photovoltaic (PV) and battery storage system	Government agencies, academics/researchers, recyclers, consultants, and manufacturers	Battery storage system related to system	Online (sent via email) and paper survey	57	Location of work, position, type of organization, and their years of experience	Cluster analysis and descriptive statistic with SPSS
This study	Australia	Disposal and recycling behavior	Individuals	Household handheld batteries (size specification: AA, AAA, C, D, 9 V, and button cell-type batteries)	Online and paper survey	400	Gender, household size, income, level of education, and age	Chi-square test, Binary logistic regression. Correlation and bivariate analysis for model validation using SPSS

Sun et al. (2015) performed a survey among residents at Dezhou City and Zibo City in Shandong Province regarding spent battery collection and recycling considering, socio-economic characteristics, consumers’ behavior on waste battery collection, awareness about harmful impacts of chemicals, knowledge about related government policies, perception on waste battery collection, and willingness to pay for a deposit refund system. Using the Grey Verhulst model (GVM) as a predictive modeling approach, Gu et al. (2017) conducted a Chinese nationwide online survey understanding ownership, replacement, and recycling-related behavior among consumers on lithium batteries derived from laptops, tablets, mobile phones, and digital cameras. Chen et al. (2017) surveyed all stakeholders associated with e-bike batteries in Xuzhou, China, to understand the dynamics (role and actions) across the waste battery recycling networks. By including a questionnaire on battery lifespan of lead-acid batteries, disposal behavior, recycling price, awareness, socio-economic information, and WTP for recycling. Tian et al. (2015) conducted a survey study on Beijing residents. Tarasova et al. (2012) assessed willingness to participate in the WB collection system, preference over disposing of the batteries, and awareness of adverse environmental impacts of the batteries among residents of Moscow and Krasnodar Krai city in Russia. In Japan, using a consumer questionnaire survey, Asari and Sakai (2013) found that when small electronic products are disposed of, 70% or more of the batteries (small sized) were not removed. Hansmann et al. (2006) proposed a contextualized model for recycling behavior considering the Swiss population for the design of public intervention strategies to enhance participation in the recycling system. Using a bivariate probit model, Arbués and Villanúa (2016) determined factors effecting individuals’ WB recycling behavior.

From the Australian context, only the study by Salim et al. (2019) was performed on WB management-related issues, which was more focused and integrated with waste solar PV and energy storage systems. In the study, the authors conducted phone interviews, email, and postal surveys on various stakeholders to assess their perceptions (drivers, barriers, and enablers) of the system. The authors concluded that there is a need for further research on consumers’ WTP for de-installation and collection models of waste battery storage and solar PV system. Unavailability of regulations and lack of awareness of safe disposal options were the significant barriers.

From the in-depth literature review, it is seen that very limited research has been performed globally as well as in the Australian context, by conducting a survey on consumers around WB disposal and recycling behavior, especially focusing on HHB. This is the first systematic study in this regard that performs statistical analysis using chi-square test of independence and binary logistic regression to identify significant association and predictive probabilities of the respondents selecting specific outcomes (i.e., preferred method of WB recycling, reasons why battery was not recycled). The results would be useful in designing a WB collection and recycling system for specific target groups, most importantly would provide valuable insights to waste management authorities for system refinement and optimization. The main research questions of this study are as follows:

1. What is the level of awareness among consumers regarding the harmful impact of waste batteries, knowledge on current information sources available on battery recycling, and perceived roles and responsibilities of a battery recycling system?
2. What is the current usage and disposal pattern of various types of batteries?
3. What are the consumers’ preferred locations for WB recycling and if batteries are not recycled what were the reasons?
4. What are socio-economic variables associated with the WB disposal and recycling behavior?

### Methodology

The survey was performed to investigate the current state of consumer awareness and behavior around WB disposal and recycling in Sydney, NSW, Australia. The questionnaire was prepared based on recommendations by Alreck and Settle (1995) and previous waste management studies by Islam et al. (2016), Islam et al. (2020a), Islam et al. (2020b), Pérez-Belis et al. (2015), and Bovea et al. (2018).Lately, Islam et al. (2021) reported that family size, age, income, gender, level of education, occupation, and type of residence were the main socio-economic variables responsible for e-waste disposal behavior. E-waste is directly connected with the types of batteries considered in this study, and except for the last two, all other variables were included in the study. From Table 2, it is also seen that types of battery use, method of disposal, recycling pattern, and wiliness to pay were comment aspects in this spectrum of research which are all included in this study. The questions related to the survey are presented in Table A1 in the Appendix.

The survey was administered using a combination of paper and online formats between March and April 2019. A pilot study with 20 participants was undertaken to check the appropriateness of survey questions and avoid misunderstandings. In addition to the abovementioned studies, papers highlighted in Table 2 were consulted for the formulations of the questions. The scope was restricted to household handheld batteries, such as AA, AAA, C, D, 9 V, and button cell-type batteries, as these dominate the Australian market (O’Farrell et al. 2014). The questionnaire consisted of the following elements:

1. Information related to respondents’ socio-economic information
2. Types of battery use (battery usage pattern)
3. Level of awareness and perception on roles and responsibilities of waste battery recycling
4. Disposal behavior in terms of places where the waste battery is discarded (current state), preferred method of disposal (from appropriate recycling perspective)
5. Recycling behavior in terms of point of recycling, information sources and reasons not to recycle, preferred ways of getting incentives, willingness to pay for recycling

A survey size of 385 samples was targeted to achieve a representative sample of the Sydney population, based on the following equation from Kotrlik and Higgins (2001) and the population of Sydney in June 2020, 5.73 million (Population.net 2020):

1 $$\mathrm{n}=\frac{{(\mathrm{t})}^{2}*(\mathrm{p})(1-\mathrm{p})}{{(\mathrm{d})}^{2}}$$

where *n* is the sample size, *t* is the *z* value (*t* = 1.96 for a 95% confidence level), *p* is the percentage of respondents who selected a specific choice (*p* = 0.50), and *d* is the confidence interval or margin of error (*d* = 5%).

A total of 400 survey responses were obtained from 450 participants that were approached both online and face-to-face. This aligns with the recommendation of Leedy and Ormrod (2013) that a sample size of around 400 is sufficient for a population size greater than 5000. The revised survey questionnaires (from the pilot survey) were distributed to randomly selected participants above 18 years old at prime locations such as train stations, universities, shopping malls, supermarkets, and libraries in the Sydney metropolitan area.

From questions A to E, socio-economic characteristics of the samples (e.g., gender, household size, income, level of education, and age) were determined, and these were considered as the independent variables for further statistical analysis. The other questions were divided into two categories. The first set of questions gathers information on the willingness to pay, disposal pathway, perceived responsibility, method of getting incentives, and general level of awareness. The second set of questions, “preferred method of disposal of waste battery” and “reasons why the battery is not recycled,” represented questions marked, M and L, respectively, attempted to identify predictive outcomes and correlation between the variables and socio-economic characteristics. In the statistical analysis, these response variables were considered “system-level decision-making aspects.” For the purposes, a statistical analysis was performed using SPSS version 19 in two separate steps.

First, a chi-squared independence test was performed to identify the statistical relationship between the first set of the (response) variables (i.e., willingness to pay, disposal pathway, perceived responsibility, method of getting incentives, and general level of awareness) and five independent variables (gender, household size, income, level of education, and age). In the second step, a binary logistic regression (BLR) was performed to identify the strength of correlation between the independent variables and two dependent variables (“consumers preferred method of disposal” and “why not battery recycled”). BLR was used as these nominal (dichotomous) variables were coded in binary form (0 if the item was not selected by the respondents and 1 if selected).

The Hosmer–Lemeshow test (goodness-of-fit statistics) has been performed for the BLR model fitting and validation exercise. The significance value (*p*-value) of the test must be higher than 0.05 to ensure model fitting (IBM SPSS Statistics 2016). If any model’s Hosmer–Lemeshow test *p*-value shows less than 0.05, then the model was revised, eliminating some of the co-variate (independent variables). Another way for model validation (model fitting) is by performing the effect size measurement. There are two ways of conducting effect size measurement, (i) by standardizing the difference between two means and (ii) by correlating independent variable classification and the individual scores on the dependent variable, and this process is called “effect size correlation” (Becker 2000). In this study, the second approach has been taken for the measurement. Details of the effect size measurement techniques have been followed the procedure mentioned in the reference of Menard (2000), Menard (2011), and Tabachnick et al. (2007). The procedure entails correlating the predicted probabilities of target group membership with the actual group membership of the independent variable. In the analysis, the predicted probability of selecting one of the options of dependent variables was correlated with the independent variables (e.g., age, income, and others). This bivariate analysis is also performed in SPSS. The analysis results come with the value of “r,” representing the correlation between the predicted probability of the individual sample and the selected dichotomous variables. The predicted probabilities of the sample can be generated using the BLR analysis (considering the independent variables), which then correlated with a dependent variable. Cohen (2013) determined the estimated value of *r* for three types of effect size, small (*r* = 0.1), medium (*r* = 0.3), and large (0.5). According to these classifications, the effect size for the models was assessed.

## Results

### Characteristics of the sample

Respondents were 52% male and 48% female. Thirty-three percent were aged 18–30, 24% aged 31–40, 21% aged 41–50, and 22% were over 50. In terms of education, 86% of respondents held a university degree (or were undertaking one) and 11% had undertaken other post-high school studies. The most common household size was 3–4 people (57%), followed by 1–2 people (31%) and ≥ 5 people (12%). Household income per month (AUD) was most commonly in the range $3000–5000 (34%), with 25% having a household income of less $3000/month, and 41% having more than $5000. The median age of survey respondents was similar to that for Greater Sydney overall (36 years), but education levels were much higher than the 28% of Sydneysiders who hold a university degree, and the median income was lower than the Greater Sydney median of $7604/month (Bureau of Statistics 2020). A similar proportion of respondents (higher responses from male than female), in terms of gender was observed in the study of Sun et al. (2015).﻿ 

### Descriptive statistics and Chi-square independence test

**Fig. 3:**
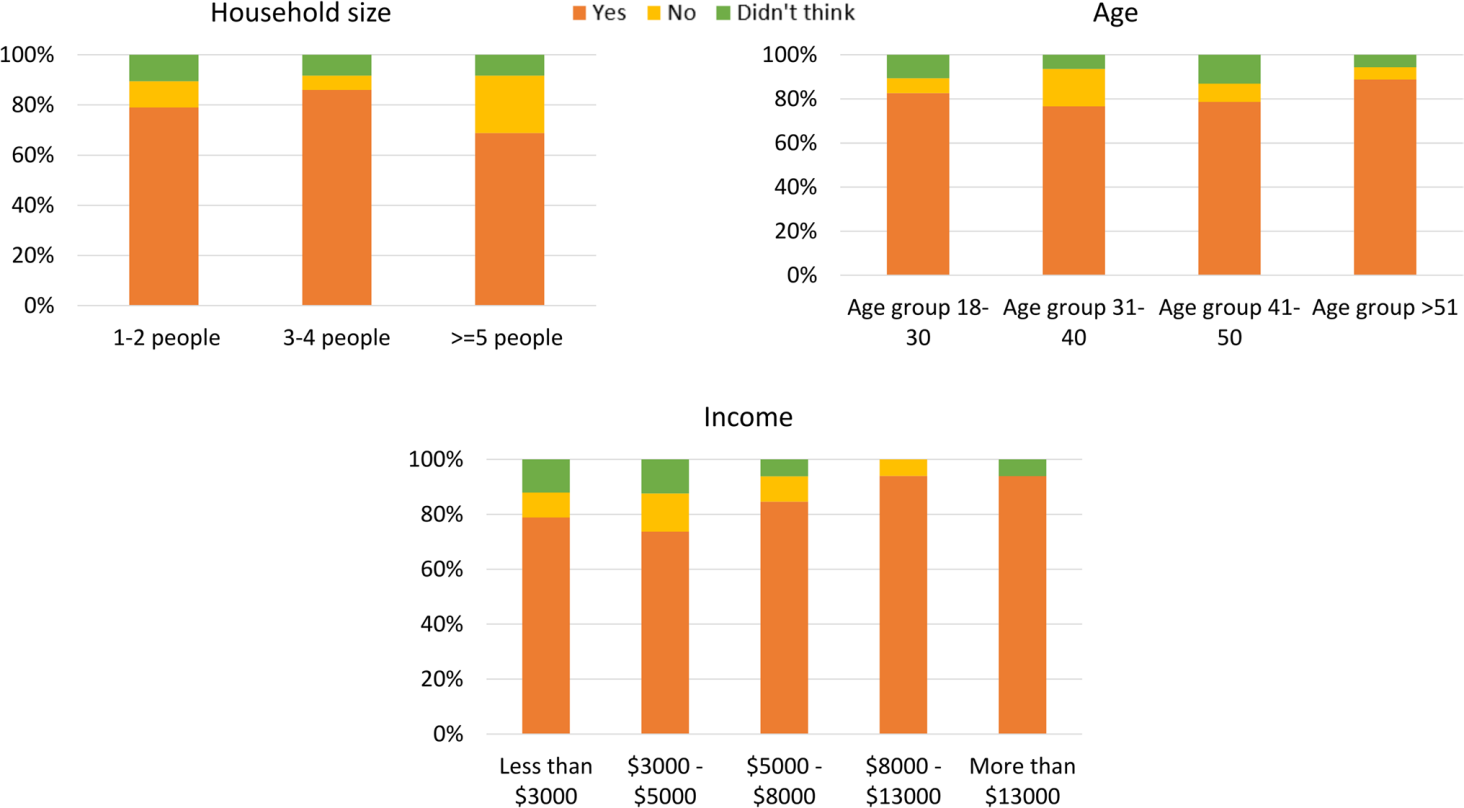
Characteristics of the respondents on level of awareness

**Fig. 4:**
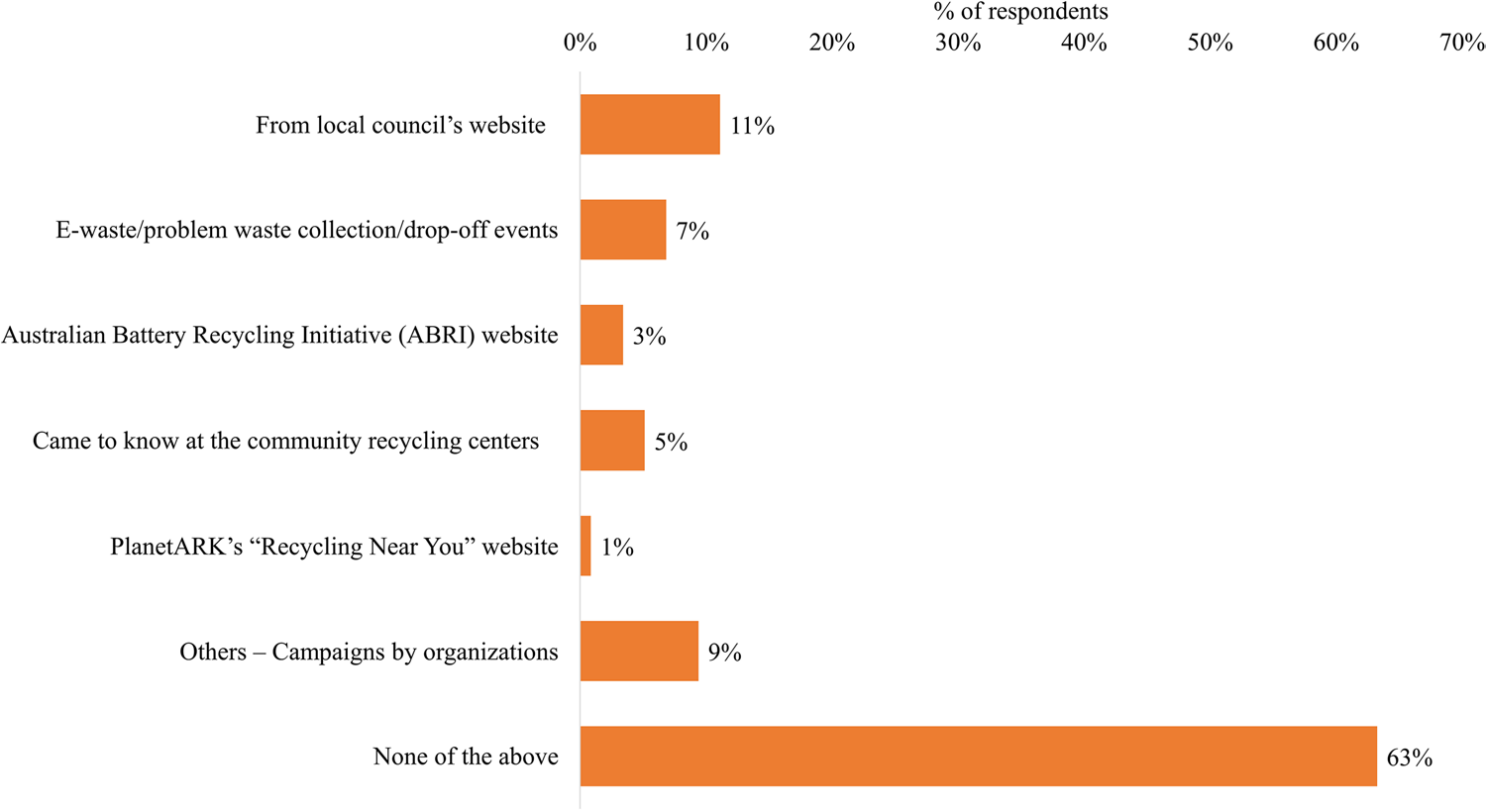
Information received by the respondents from various sources

**Fig. 5:**
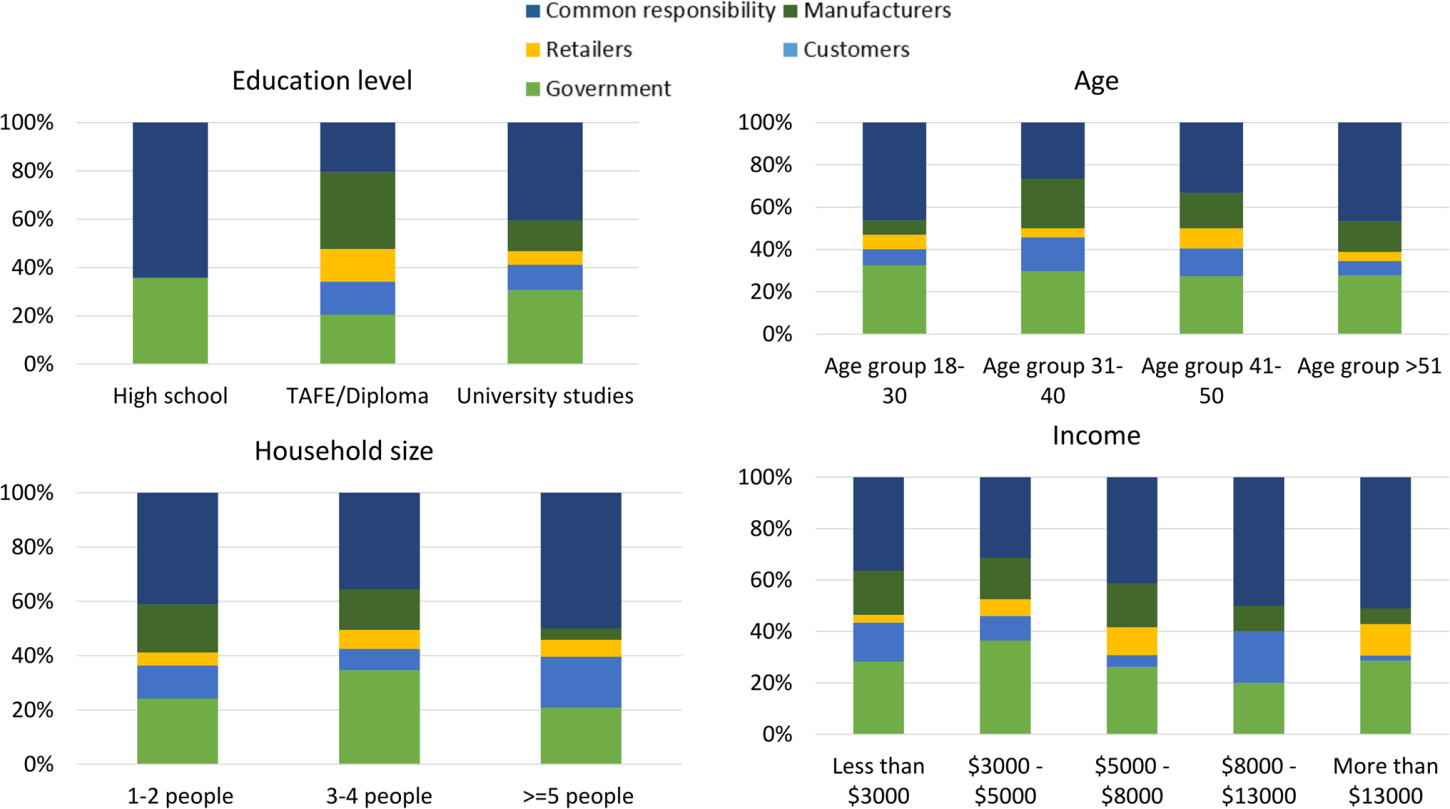
Characteristics of the respondents for perceived responsibility

**Fig. 6:**
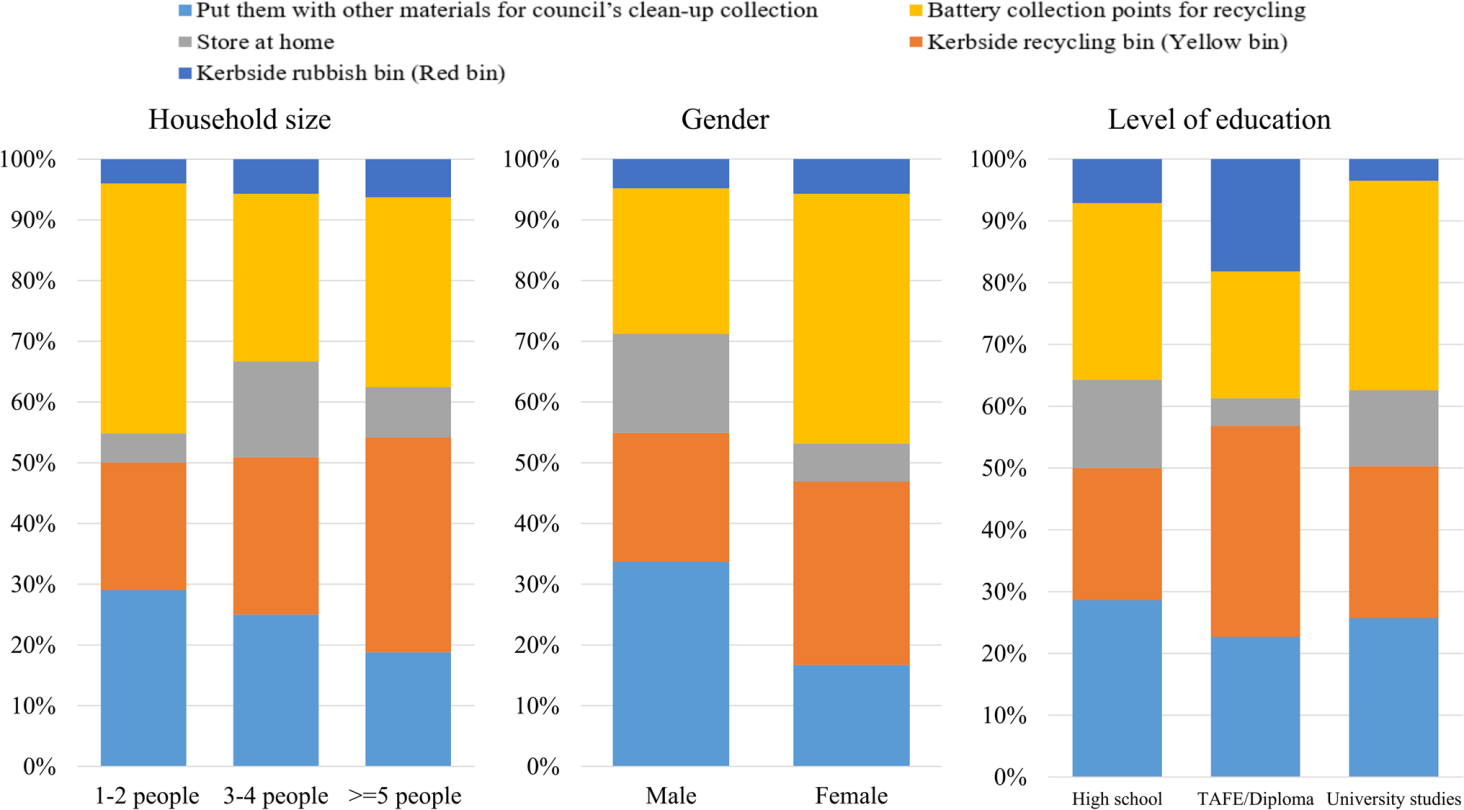
Characteristics of the respondents for methods of waste battery disposal

**Fig. 7:**
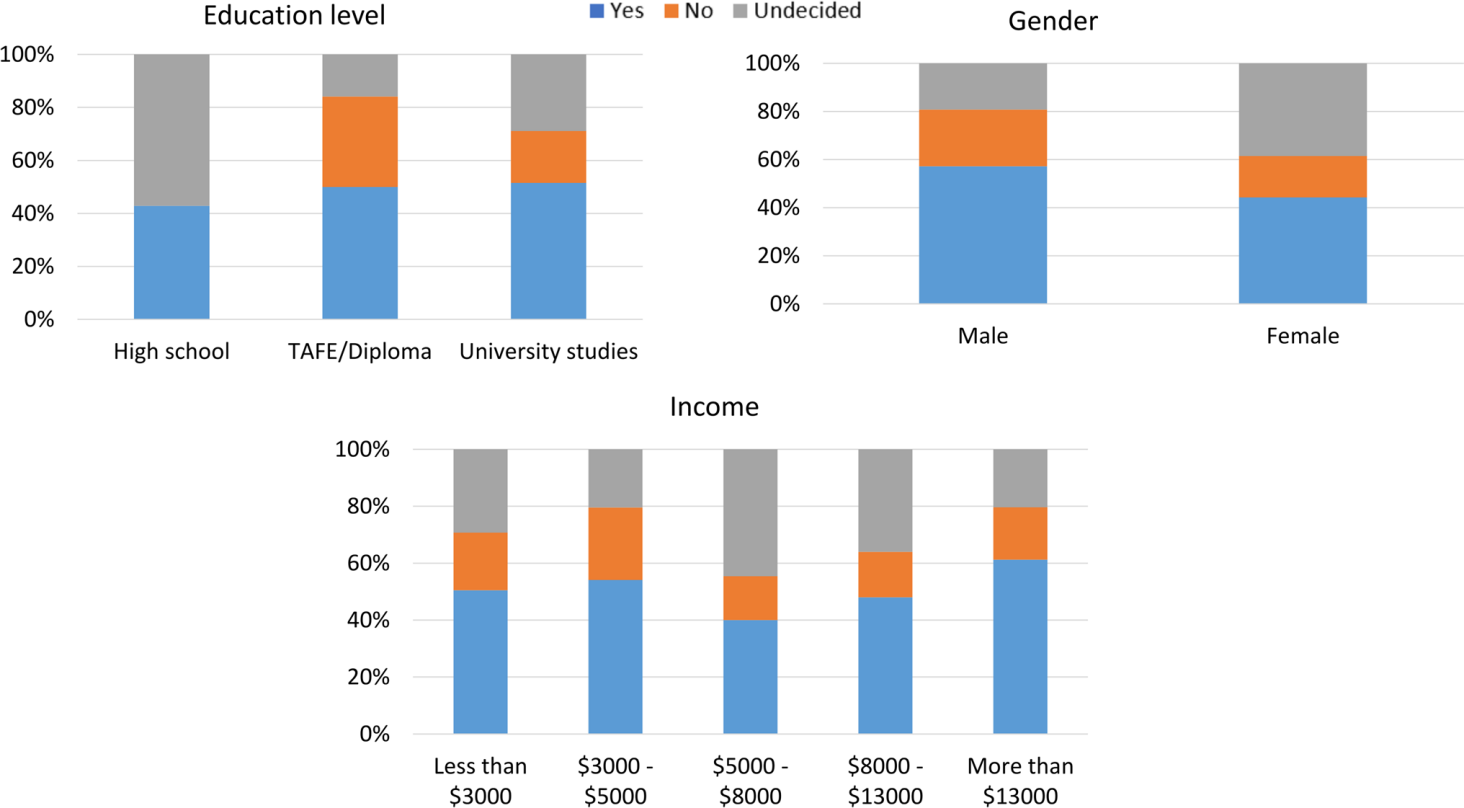
Characteristics of the respondents for WTP

The results of the chi-squared analysis are shown in Table 4. For each test, the null hypothesis was that the answers given by respondents (i.e., on willingness to pay, disposal pathway, perceived responsibility, method of getting incentives, and general level of awareness) were independent of the respondent’s category (i.e., that there was no relationship between the answers they gave and their gender, household size, income, level of education, or age). A *p*-value of 0.05 was applied for significance, meaning that if the *p* value was less than 0.05, the null hypothesis was rejected, and it was concluded that there was a relationship between the answers given and the respondent’s category. Relations related to the level of awareness, information sources, perceived responsibility, method of battery disposal, and WTP with various socio-economic variables are shown in Figs. 3, 4, 5, 6, and 7, respectively.

**Table 4:** Chi-square independence test of variables

	Gender		Household size		Income		Level of education		Age	
Variable	*χ*^2^	*p*-value	*χ*^2^	*p*-value	*χ*^2^	*p*-value	*χ*^2^	*p*-value	*χ*^2^	*p*-value
Willingness to pay (WTP)	18.318	**0**	9.162	0.057	17.205	**0.028**	13.679	**0.008**	5.181	0.521
Disposal pathway	32.579	**0**	18.285	**0.019**	9.56	0.889	22.139	**0.005**	161.858	**0.000**
Perceived responsibility	5.641	0.228	16.705	**0.033**	35.492	**0.003**	26.508	**0.001**	27.048	**0.008**
Method of getting incentives	1.7111	0.634	9.381	0.153	16.1511	0.169	9.708	0.138	9.306	0.41
General level of awareness	2.306	0.316	15.039	**0.005**	19.693	**0.012**	3.882	0.422	13.212	**0.04**

Bold numbers refer to significant correlations between the variables (*p*-value ≤ 0.05)

#### Level of awareness and information sources

Most respondents (82%) believed that waste batteries have a negative impact on the environment and human health if not properly collected and recycled, while 9% thought they were not harmful and the other 9% had not thought about this previously. Awareness level differed by household size, income, and age. Participants who were older, had higher incomes, and were from smaller household sizes were more likely to have a higher level of awareness (Fig. 3). Among the survey respondents, lack of knowledge was particularly an issue for those who were younger, had lower incomes, and had larger household sizes. Environmental concern was one of the significant factors among Spanish residents for battery recy-cling (Arbués and Villanúa 2016). Environmental education is the key to future recycling practice (Arbués and Villanúa 2016). To increase overall recovery rates, awareness-raising programs could also be implemented at battery collection points in other public facilities, such as libraries, schools, and community recycling centers. In the Australian high school curriculum (especially in the years 11 and 12), three different units such as (1) “Introduction to Earth systems,” (2) Earth processes – energy transfers and transformations, and (3) Living on Earth – Extracting, using and managing Earth resources” encompass various subject contents and learning outcomes. More specifically, Unit 3 covers earth resources extraction, use and management, and associated waste management of renewable and non-renewable resources-related topics (Australian Curriculum 2021). However, in the unit description, the in depth waste management-related focus is currently missing, including the high school curriculum and the various environmental and human health impacts of improper disposal (e.g., heavy metal contamination in landfills). Plastics, electronic, organic food, and textile waste should be highlighted along with the battery waste. Issues around battery waste are critical because many young adolescents’ everyday items, such as gaming devices, electronic toys, drones, and remote controls, are run by small handheld batteries. Their adequate knowledge and awareness are specifically required to develop a sustainable WB collection and recycling system. In a study by Ergül and Çaliş (2017), it was found that high school students in Turkey were not aware of the type and source of electronic waste, and it was recommended that teachers should also be mindful of specific waste-related knowledge. Thus, educational institutions and high school curriculum could essentially play a critical role in such instances. Eneji et al. (2019) concluded that environmental education strongly affects positive environmental attitude, behavior, and perception towards posi-tive waste management. Hasan (2004) recommended that waste education be mandatory in the school curricula from kindergarten to high school levels.

When asked about sources of information on WB collection and recycling (Fig. 4), 63% said they did not receive information from any source. Of the information sources listed, local government was most commonly selected (11% of respondents), followed by campaigns (9%), drop-off events (7%), and visits to community recycling centers (5%). Notably, only 1% of respondents selected the website “Recyclingnearyou.com.au,” run by the not-for-profit organization PlanetARK. It is Australia’s the most comprehensive information database of recycling locations and services. In response to a follow-up question about this website, 4% of respondents said they used it, compared with 23% that knew of it but did not use it and 73% that did not know of it at all. Similarly, there was a low level of knowledge about the Australian Battery Recycling Initiative (ABRI) (i.e., with only 3% of respondents listing this as an information source). In the study of Sun et al. (2015), non-governmental organizations (NGOs) and the government were the two agencies from where information was less disseminated among respondents in China. However, not-for-profit firms are capable to reduce waste generation (Pirani and Arafat 2014). Unlike above, local councils were the most cited source, from where consumers received infor-mation on WBs recycling. Thus, they could play an increased role in raising awareness among con-sumers by providing information stickers on red and yellow bins, and leaflets or handouts during council clean-up collections and special events with the strategic partnership of ABRI, Planet ARK, and proposed scheme members. In Switzerland, the battery-recycling organization INOBAT sup-ports municipalities and collection centers by providing a battery recycling logo, which is embedded in a printed matter to sensitize and deliver information to the residents of a council (INOBAT 2019a). Information campaigns were identified as a potential pathway for better WB collection-related outcomes (Tarasova et al. 2012).

#### Perceived responsibility

In the survey, consumers were asked who should be responsible for battery collection and recycling in Australia. The most widely selected option (42% of respondents) was “common responsibility,” while 29% identified this as the role of government, followed by manufacturers (14%), customers (9%), and retailers (6%). Waste management is a multi-actor, multi-faceted task, and needs the par-ticipation of all actors in the system (Pires et al. 2011). Perceived responsibility differed by house-hold size, income, level of education, and age. Respondents from medium-sized households (3–4 people) were more likely to say that battery recycling was a responsibility of the government than respondents from other household sizes. Participants with incomes of $3000–5000/month and par-ticipants aged between 31 and 40 were also more likely to say this is the responsibility of the gov-ernment. TAFE/diploma qualification holders were more likely to believe that manufacturers are responsible for WB management (Fig. 5).

#### Battery use and disposal methods

Forty-seven percent of participants indicated that they used single-use (primary) batteries, while 29% said they used rechargeable (secondary) batteries and 23% used both. Single-use batteries are generally disposed of without recycling. Regarding disposal methods, 30% of respondents said they took WBs to battery collection points. One probable reason could be re-spondents were coming from higher-than-average education levels in Australia’s largest city, where access to collection sites is likely to be greatest, while 27% disposed of them in red (general waste) bins and 24% disposed of them in yellow bins, which are designed for recyclable paper, plastics, cardboard, and aluminum cans rather than WBs (City of Sydney 2022). “Disposal with household trash” was a common practice among Russian residents (Tarasova et al. 2012). A similar reason was also identified by Arbués and Villanúa (2016) mentioning that Spanish-born, high-income owner, and city dwellers who came across environmental campaigns showed a positive battery recycling attitude. Many of the batteries are attached to small e-waste items such as toys, remote control, which are currently unregulated in Australia (Islam and Huda 2020a). Gu et al. (2017) identified that the exclusion of waste LIBs from the e-waste collection system in China reduced the overall recycling rate in China. In Japan, this misplaced battery stream represents 10–40% of the WB and limited knowledge and awareness about laws and regulations were the major inhibitors of small-size battery waste collection (Asari and Sakai 2013).

Disposal pathways differed by gender, household size, and education level. Females were more in-clined to dispose of WBs at recycling points than males. In terms of household size, small house-holds (1–2 people) and medium households (3–4 people) were more likely to utilize recycling points, while participants in large households (≥ 5 people) were more likely to dispose of WBs in their yellow bins. Participants with university degrees were more likely to select recycling points as their disposal pathways than those with high school qualifications only. Respondents with TAFE/diploma qualifications identified kerbside yellow recycling bins as their most-preferred dis-posal route. Detailed characteristics of the respondents in relation to waste battery disposal paths are shown in Fig. 6. In the sample of Hansmann et al. (2006), older participants were more inclined to appropriate disposal behavior; however, statistically, the effect was very small. 

These bin-level disposal patterns are also indicating that batteries are not properly recycled. In the present survey, around 14% of the respondents stored WBs at home. With a subsequent face-to-face conversation with the respondents, it revealed that WBs were typically stored for 1 month before disposal. Five percent of the respondents put their waste batteries out for collection at “council clean-ups.” Most material collected in red bins goes to landfill (City of Monash 2021). “Stored at home” or “thrown away as household waste” were the two options selected by the Chinese residents (Sun et al. 2015). 

Sixty-four percent of the respondents “never recycled” their WBs, and the WB stream went to vari-ous destinations. The survey results were consistent with previous studies showing that the majority of WBs in Australia are not recycled (ABRI 2017, O’Farrell et al. 2014). Respondents who dis-posed of WBs in waste collection bins were asked why they did so. The majority (53%) answered that they did not know where and how to recycle WBs, while 23% said they did not have time and 6% said that recycling points are too far from their households. A small percentage (6%) thought that batteries could not be recycled. The results indicate that the two most important factors that inhibited respondents from participating in battery recycling: (1) lack of knowledge and information on the recycling system and (2) convenient collection services. These were also the findings of Gu et al. (2017)’s study in China. The combined efforts of councils, NFP, and other public facilities may help reduce the knowledge gap amongst consumers who dispose of WBs inappropriately because they “don’t know how and where to recycle.” Specific information could be provided to consumers at the point of kerbside recycling collection (i.e., on yellow bins or in associated pamphlets). This information could inform residents that materials other than those designated for yellow bins are not being sorted, and unwanted material goes to landfill. For collection, one option that could be applied in Australia is to place a battery collection bag at the point of purchase. This method has been successfully employed by INOBAT in Switzerland (INOBAT 2019b). A free mobile phone app could also help consumers to find the nearest WB collection points. Such type of model was applied for textile waste in New York, USA (Ellen MacArthur Foundation 2022). An interactive app-based approach may be easier and more convenient than searching for static web-based infor-mation (such as presented on the “Recyclingnearyou.com.au” website). The overall number of col-lection points in Australia also needs to increase significantly. It is estimated that the total number of WB collection systems in Australia is 619 out of which 500 collection points are run by ALDI (e.g., one of the supermarket retailers) across the country. However, there is a clear lack of comprecomprehensive information on the number of collection points available for WB across the country which might also be the reason for the low recycling rate. In Switzerland, there are more than 11,000 collection points, including retail to specialist outlets and department stores, post offices, kiosks, and filling-station shops (INOBAT 2018). The development of the reverse logistics network system and the role of extended producer responsibility were found as two main enablers for battery recycling in China (Chen et al. 2017).

#### WTP and incentives

When asked if they would be willing to pay an additional price of 20% (i.e., AUD 0.10 for an AAA EVEREADY battery) to fund the recycling scheme, 53% of respondents said yes, 25% were unde-cided, and 22% answered no. Willingness to pay (WTP) differed by gender, income, and education level. Participants who were male, had higher incomes, and had higher education levels (compared to high school graduate) were more likely to indicate that they were willing to pay for battery recy-cling) (Fig. 7). Notably, the preferred incentive method showed no statistically significant relation-ships with any of the independent variables tested using chi-squared analysis. In terms of WTP, it was found that a medium-size family (3–4 people) was the most willing to pay additional fees (i.e., advanced fees) for battery recycling.

This study revealed that most consumers were willing to pay more to enable a deposit-return scheme. However, further investigation is required to calculate an appropriate deposit amount. In a recent study in the USA, Arain et al. (2020) found that survey participants were willing to pay $2.90 to recycle a battery (not specified what type of battery). In Sun et al. (2015)’s study, respond-ents were willing to pay 0.39 Chinese Yuan/unit of battery (15.6% of the sales price for the deposit-refund system). In the present study, only 53% of respondents were willing to pay 20% more for batteries to enable such a scheme, but a willingness to pay may increase if costs were lower or awareness of negative impacts was increased. Tian et al. (2015) found that education level was not the critical factor for consensus towards WTP; rather, it is the environmental protection training at school which was important. Factor towards WTP varies substantially, for example, Tian et al. (2015) found that recycling plants located outside and treating the majority of the city’s battery waste would receive more financial support from the residents. When asked to select their preferred form of incentive for disposing of batteries at recycling points, around 57% of respondents pre-ferred a hypothetical “old-for-new” scheme whereby they give their old batteries to a retailer to get new batteries. Another 18% stated that they would prefer vouchers to shop at the supermar-ket/retailer where WBs are collected and 13% preferred a cash incentive. The final option, receiving a voucher but donating it to contribute to the costs of running the scheme, was the least preferred (12% of respondents). One of the innovative solutions could be a “battery bag” that could potential-ly be used to deposit batteries into a return machine, which would then compute the amount that a customer would be entitled to receive based on weight. Customers in this survey were willing to participate by returning WBs for collection and paying more for batteries due to additional fees dur-ing purchase, if they were given clear information of where to return batteries, convenient collection sites, and incentive such as old-for-new, vouchers, or cash. This system can be operationalized in a future “battery stewardship scheme” under retailed-based collection mechanism, which would also reduce the overall logistical costs of WB recovery systems.

#### Recycling behavior

**Fig. 8:**
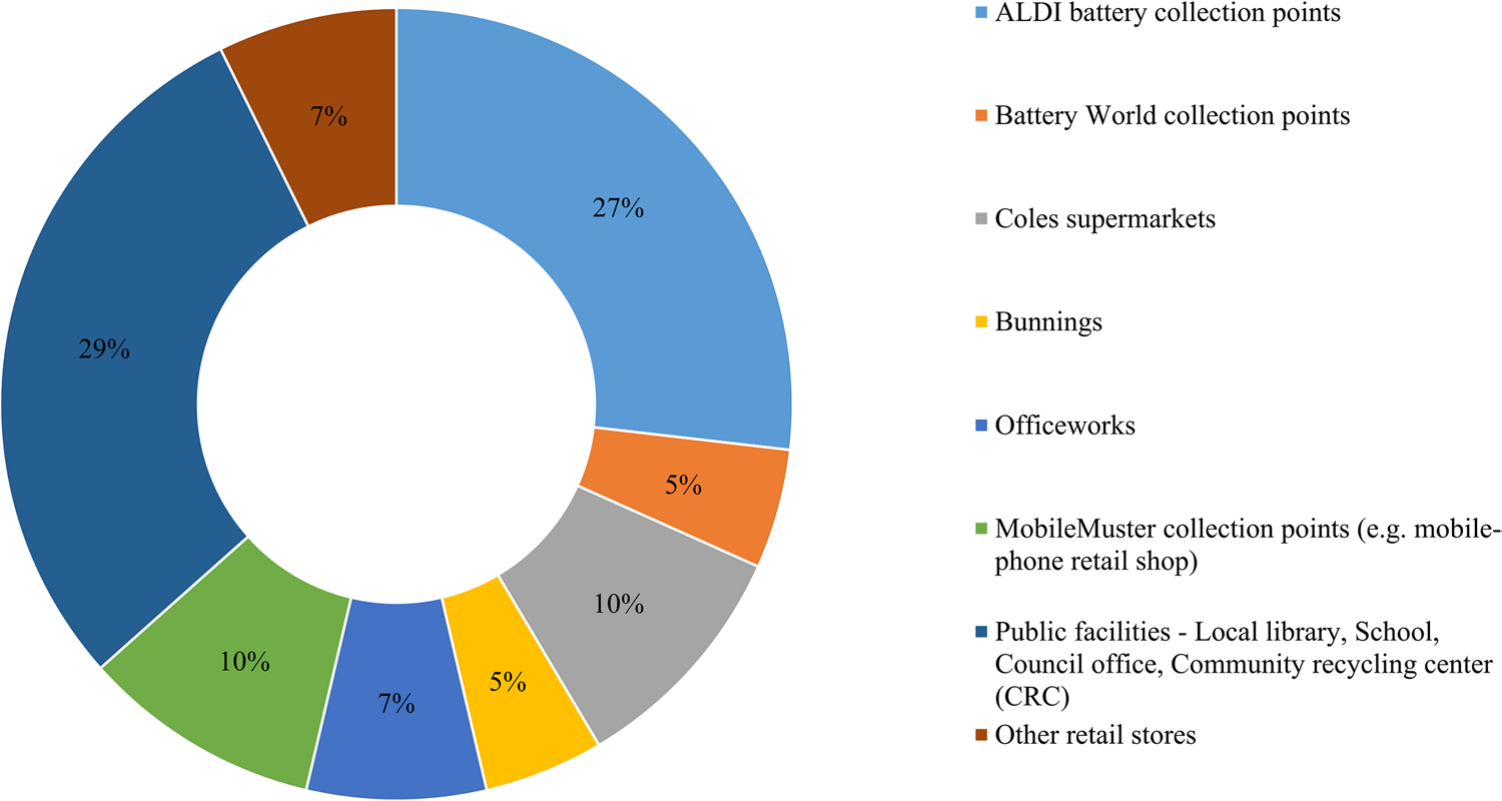
Locations where respondents dispose of batteries for recycling

Of those respondents that said they recycle WBs, the most common locations (Fig. 8) were super-market retailer such as Aldi (27%). Retailer-based collection systems have been found to be efficient and effective in other countries, particularly when there is competition among retailers to receive WBs due to incentives provided to retailers (Guo et al. 2018). Retailers were identified as a vital element for a successful reverse supply chain or waste collection network in Australia (ANZRP 2022). Public facilities were used by 29% of respondents who recycled WBs, with other recycling locations including Officeworks (7%), IKEA (7%), Bunnings (5%), and Battery World (5%). According to PLANETARK’s Recycling Near you (2021), ALDI, Battery world, and Office works are the major retailers that collect WB in Australia. Mobile Muster collection points, which are generally located at mobile phone retailers to collect rechargeable batteries from waste mobile phones, were selected by 10% of respondents who recycle WBs. Around 10% of the respondents selected Coles as the other retailer where they think they recycled their WBs. Coles signed an agreement with the Battery Stewardship Council (ColesGroup 2021), and WB collection bins are available to some participating outlets and for employees at the workplace. Participants were asked to select their preferred WB collection systems from four possible options. The most preferred option (chosen by 39% of respondents) was a “Return and earn” deposit return scheme similar to that used for plastic bottles and aluminum cans. In the collection scheme, customers get $0.10 for each bottle or can be deposited at a local collection center, which is paid by customers in advance while purchasing. Sun et al. (2015) proposed to apply the scheme for spent battery collection in China, giving an example of Sweden, where the scheme is widely used for beverage containers collection. The second most-preferred option (27% of respondents) was for batteries to be collected at super-markets/retail stores within the proximity of consumers, which is an existing option already being utilized by a majority of the respondents who recycle their batteries. Curbside recycling bins for WBs were preferred by 26% of respondents and 8% preferred door-to-door collection. In the following section, a binary logistics regression analysis was performed to understand critical socio-economic aspects of recycling behavior among consumers.

### Binary logistic regression (BLR) analysis 

The results of the binary linear regression (BLR) analysis are shown in Table 5 and Table 6. Applying a *p*-value of less than 0.05 for significance, these results show that the method “collected at supermarket/retail store with higher proximity” (Table 5) has a positive predicted relationship with income (i.e., participants with household incomes of “less than $3000/month” and “$3000–$5000/month” were more likely to select this option than they were to choose the other options, such as Return and Earn scheme), while household size has a negative predicted relationship with this method, which means that larger household size is less likely to select “collected at supermarket/retail store with higher proximity” than other methods. On the other hand, a newly proposed collection method similar to the “Return and Earn” bottle/can collection method is related to education level (i.e., respondents with higher education levels were more likely to select this option than they were to select other options). The other two methods, the “kerbside recycling method” and “door-to-door collection” do not have any significant relationship with the independent variables. Age was the significant predictor of recycling behavior in the study of Arbués and Villanúa (2016) and (Hansmann et al. 2006). One probable reason for identifying age as a predictor because in the present study, recycling behavior was analyzed further categorical level.

**Table 5:** Model coefficient, *p*-value, goodness-of-fit and effect size of binary logistic regression mode, considering “preferred method of disposal” and independent variables

Parameter	BLR model coefficient	BLR *p*-value	Hosmer and Lemeshow test (goodness-of-fit) for BLR, *p* value	Effect size measure
Kerbside recycling bins for batteries				
Gender	− 0.061	0.779	0.441	*r* = 0.068 *r*^2 = 0.00462
Age	0.07	0.454		
Education	− 0.134	0.556		
Household size	0.154	0.386		
Income	− 0.036	0.676		
Door-to-door collection				
Gender	− 0.082	0.806	0.115	*r* = 0.16 *r*^2 = 0.0256
Age	− 0.074	0.601		
Education	0.393	0.355		
Household size	0.431	0.108		
Income	0.188	0.132		
Disposal at supermarket/retail store with higher proximity				
Gender	− 0.433	0.056	0.319	*r* = 0.194 *r*^2 = 0.03763
Age	− 0.084	0.388		
Education	0.215	0.398		
Household size	− 0.533	**0.005**		
Income	0.198	**0.025**		
A system that can return deposit as for “Return and Earn” bottle recycling				
Gender	0.247	0.225	0.748	*r* = 0.146 *r*^2 = 0.02131
Age	− 0.02	0.823		
Education	− 0.468	**0.038**		
Household size	0.04	0.808		
Income	− 0.148	0.067		

Bold numbers refer to significant correlations between the variables (*p*-value ≤ 0.05)

**Table 6:** Model coefficient, *p*-value, goodness-of-fit and effect size of binary logistic regression mode, considering “why the battery is not recycled” and independent variables

	BLR model coefficient	BLR *p*-value	Hosmer and Lemeshow test (goodness-of-fit) for BLR, *p* value	Effect size measure
Recycling points are far from my household				
Gender			0.841*	*r* = 0.140 *r*^2 = 0.0196
Age	0.025	0.848		
Education				
Household size	0.25	0.489		
Income	0.285	**0.012**		
I do not think battery can be recycled				
Gender	0.126	0.783	0.298	*r* = 0.242 *r*^2 = 0.058564
Age	0.159	0.411		
Education	− 0.703	0.052		
Household size	0.89	**0.016**		
Income	− 0.302	0.125		
Do not know how and where to recycle				
Gender	0.003	0.989	0.121	*r* = 0.288 *r*^2 = 0.082944
Age	− 0.055	0.55		
Education	1.092	**0**		
Household size	− 0.251	0.146		
Income	− 0.236	**0.005**		
I do not have time disposing of them				
Gender			0.069*	*r* = 0.083 *r*^2 = 0.006889
Age				
Education				
Household size				
Income	0.153	0.099		

Bold numbers refer to significant correlations between the variables (*p*-value ≤ 0.05). Hosmer and Lemeshow test is for goodness-of-fit for the models. If the *p* value of the test is higher than 0.05, then the model is fitted. *Results of the revised model

The BLR analysis for the question “why battery is not recycled” (Table 5) showed that the level of education among the respondents has a significant relation with several factors, such as “Recycling points are far from my household” (i.e., respondents with high school qualification and university degree), “don’t know how and where to recycle” (i.e., high school qualification), and “don’t have time to recycle” (i.e., participants with university qualifications). On the other hand, male respondents were significantly more likely to indicate that they do not have time to dispose of waste batteries than they were to select other reasons. Medium-sized households (3–4 people) were significantly more likely to select the reason “I do not think battery can be recycled” than they were to select other reasons. The income level of the participants also showed a significant correlation to the answer “don’t know how and where to recycle,” with participants with income $3000–$5000 per month choosing this answer more frequently than other options.

### BLR model validation

In the BLR models, Hosmer and Lemeshow test (goodness-of-fit) was performed, and it is seen that all the analyses (i.e., “preferred method of disposal” as dependent variables and socio-economic variables) showed the goodness-of-fit (e.g., *p* values greater than 0.05) (Table 5). When considering “why the battery is not recycled” and independent variables, it is seen that except for the (dependent) variables “Recycling points are far from my household” and “I do not have time disposing of them,” all the models fitted appropriately (Table 6). In these cases, the models were revised, and gender and education (from the first case) and gender, education, age, education, and household size (for the latter case) were taken out from the analysis (i.e., model fitting exercise). This result also implied that age, household size, and income are correlated with the location of the household from the recycling points; income should be considered as the predicted factor in this instance. Although insignificant in terms of BLR model *p*-value identified, income was also the main parameter responsible for model fitting for the “I do not have time disposing of them” response variable. Overall, it can be said that the developed BLR qualified against the goodness-of-fit (i.e., all test *p* values are more significant than 0.05). Effect size measurement calculation also showed that the shared variation of the variables existed and ranged from small to medium effect sizes, except the assessment of the “Do not know how and where to recycle” variable with the independent variable that showed medium effect size (*r* = 0.288, *r*^2 = 0.082944). In terms of interpretation, it can be said these two variables (predicted probability of the sample that contains characterize of independent variables) and the dependent variable “Do not know how and where to recycle” had shared over 8% common variance. In other words, there was a modest correlation between predicted probabilities and actual group membership. “ < small effect size” was observed for “Kerbside recycling bins for batteries” and “I do not have time disposing of them.”

## Limitations and future research

Further research could be carried out in other metropolitan areas of Australia and with larger sample sizes to gain a more in-depth understanding of consumer behavior around WB recycling. Another research opportunity could be identifying respondents with and without children to understand their differing patterns of battery disposal and battery lifespan. Surveys of specific groups, such as a medium-sized family (3–4 people) with a monthly income of $3000–$5000 with parents falling in the range of 31–50 years old could provide a more holistic understanding of several indicators used in this study. In the BLR analysis, only two of the response variables were considered, “preferred method of disposal” and “reasons not to recycle waste batteries,” which could be expanded to other variables, such as a method of getting incentives or WTP. In this study, HHB batteries were only considered, especially batteries were with the size of AA, AAA, C, D, 9V, and button cell, excluding other emerging battery waste streams such as LIBs, and this should be considered as one of the limitations of the present study.

As it is seen from Table 1, recycling processes for different battery types vary substantially. Therefore, depending on various battery types and chemistry involved, detailed techno-economic analysis is required for assessing the economic viability and environmental sustainability of different recycling processes. Although this area is out of the scope of this paper, consumer behavior would critically contribute to the collection infrastructure and downstream recycling efficiently. Of course, lately, lithium-ion battery recycling and reuse have received substantial attention among policymakers and research organizations (Zhao et al. 2021); however, this should be expanded to other battery types.

Future studies should focus on consumers’ disposal and recycling behavior on spent LIBs. This type of battery is closely associated with the small e-waste items (e.g., mobile phones, toys, digital cameras, laptops, tablets) and many of the items are currently not covered or partially covered under waste collection schemes (Islam and Huda 2020b). A similar study could be performed, including residence and occupation as other socio-economic variables. Depending on the market size and household penetration rate, such a study could provide more insights into holistic perspectives on the Australian battery waste management system for efficient resource recovery.

## Conclusion

The survey aimed to identify consumers’ battery use and disposal behavior, awareness of issues relating to the improper disposal of batteries, preferred methods of disposal, preferred incentives, general understanding of the roles and responsibilities of different stakeholders in the system, and willingness to pay for battery recycling. This study provides valuable insights by understanding current consumers’ disposal and recycling behavior in general and at a socio-economic level that could be used as an input for future waste battery recycling systems. From the research perspectives, limited research in the consumer behavior around WB was performed worldwide, and this study presented a first systematic study on the issues in the Australian context. Weight-based battery beg, mobile app, strategic partnership, on Australian high school education curriculum development, and emulation of bottle recycling system for WB, and old-for new incentive system are some of the potential paths highlighted in this study.

Despite respondents having a general awareness of the negative impacts of improper disposal of batteries, this knowledge did not translate into sustainable practice. Results of the statistical analysis showed that family size and income were significantly associated with knowledge and awareness, which need to improve. On the other hand, the convenience of the collection system and improper disposal-related issues (time limitations, lack of knowledge about collection points, proximity of recycling center) were significantly associated with gender and level of education of the participants, respectively. Medium income families, the large household size preferred retailer-based collection system while highly educated individuals preferred “Return and Earn”-type mechanism. To improve the future performance of the waste battery recovery and recycling sector in Australia, critical elements identified by this study were local councils and not-for-profit initiatives to raise awareness at the household and institutional level, developing a retailer-based collection and recovery network, introducing “old-for-new” incentives, and deposit-return system, and consideration for collection of batteries alongside small e-waste.

## Data Availability

The datasets used and/or analyzed during the current study are available from the corresponding author on reasonable request.

## Appendix

**Table A1:** Questionnaire survey form for battery disposal and recycling

Factor	Response
A. Please indicate your gender.	☐ Male ☐ Female
B. Please indicate your age.	☐ 18-30 ☐ 31-40 ☐ 41-50 ☐ >51
C. Please indicate your level of education	☐ Primary school and below ☐ High school ☐ Tafe/Diploma ☐ University studies (bachelor, Masters, Phd)
D. Please indicate your household size	☐ 1-2 people ☐ 3-4 people ☐ >=5 people
E. Please indicate the level of family income per month	☐ Less than $3000 ☐ $3000 - $5000 ☐ $5000 - $8000 ☐ $8000 - $13000 ☐ More than $13000
F. What type of portable battery do you use in your households?	☐ Primary batteries (single use) ☐ Secondary batteries (Rechargeable)
G. Do you know that improper disposal of the battery is bad for the environment and loss of resources as well. –	☐ Yes ☐ No ☐ Didn’t think about it
H. How do you dispose of your batteries?	☐ Kerbside rubbish bin (Red bin) ☐ Kerbside recycling bin (Yellow bin) ☐ Store at home ☐ Battery collection points for recycling ☐ Put them with other materials for council’s clean-up collection
I. For recycling, where do you take your batteries?	☐ ALDI battery collection points ☐ Battery World collection points ☐ Coles supermarkets ☐ Bunnings ☐ Office works ☐ Mobile Muster collection points (e.g., mobile phone retail shop) ☐ Public facilities - Local library, School, Council office, community recycling centers (CRCs) ☐ Other retail stores ☐ Never recycled
J. From which source, did you received information on battery recycling?	☐ From local council’s website ☐ E-waste/problem waste collection/drop-off events ☐ Australian Battery Recycling Initiative (ABRI) website ☐ Came to know at the community recycling centers ☐ PlanetARK’s “Recycling Near You” website ☐ Others – Campaign by organizations ☐ None of the above
K. How often do you use PlanetARK’s “Recycling Near You” website to find battery recycling points?	☐ I use it frequently ☐ I don’t use it ☐ Didn’t know that the website exists
L. If you don’t recycle the batteries, what are the reasons?	☐ Recycling points are far from my households ☐ I do not think battery can be recycled ☐ Don’t know how and where to recycle ☐ I don’t have time disposing of them ☐ Not applicable (in case you recycle)
M. In your opinion, what are the most convenient ways of disposing of waste batteries?	☐ Kerbside recycling bins for battery ☐ Door-to-door collection ☐ Collected at supermarket/retail store with higher proximity ☐ A system that can return deposit as if “Return and Earn” bottle recycling
N. If there is a system that gives you incentives, what is your preferred method?	☐ Old-for-new (you give retailer old batteries and get a new one) ☐ Vouchers to shop other items from the supermarket ☐ Receive vouchers and donate ☐ Cash ☐ Others (Please suggest) ……………….
O. Who should be responsible and more active in battery recycling in Australia?	☐ Government ☐ Customers ☐ Retailers ☐ Manufacturers ☐ All above
P. Are you willing to pay additional 20% ($0.1 per unit) of the actual price of a battery for recycling (for example – the current price of an EVEREADY AAA battery is $0.5/unit, which will be $0.6/unit)?	☐ Yes ☐ No ☐ Undecided
